# Comparative efficacy of medications and compound interventions against porcine epidemic diarrhea virus replication in vitro: a systematic review and meta-analysis

**DOI:** 10.1186/s12917-026-05306-0

**Published:** 2026-01-30

**Authors:** Hong Zou, Shilei Huang, Zhiping Mu, Gan Luo, Wenting An

**Affiliations:** 1 College of Animal Science & Technology, Chongqing Three Gorges Vocational College, Chongqing, 404100 China; 2 Wanzhou Center for Animal Husbandry Industry Development of Chongqing, Chongqing, 404100 China

**Keywords:** Porcine epidemic diarrhea virus, Medications and compound interventions, Systematic review and Meta-Analysis

## Abstract

**Background:**

Porcine epidemic diarrhea virus (PEDV) infection induces severe intestinal disease in neonatal piglets, resulting in substantial economic losses to the swine industry. Despite the availability of vaccines, their limited efficacy and the lack of effective antiviral treatments underscore the need for alternative therapeutic approaches. Several studies have reported the antiviral activity of various drugs and compounds against PEDV. To systematically compare the efficacy of these agents, we conducted a systematic review and meta-analysis to evaluate the inhibitory effects of various drugs or compounds on PEDV replication in vitro using cell based- models.

**Methods:**

A comprehensive literature search was conducted in PubMed, Web of Science and ScienceDirect, encompassing publications from the inception of each database to May 30,2025. Eligible studies primarily focused on the effects of pharmacological and combination interventions on PEDV replication in vitro. Changes in the tissue culture infectious dose (TCID50) served as the primary outcome measure, with standardized mean difference (SMD) used to quantify effect sizes.

**Results:**

Thirty-two eligible studies involving 41 distinct drugs and 63 effect sizes were analyzed. A random-effects model revealed an overall SMD of -12.30 (95% confidence interval: -13.64 to -10.95) for the antiviral efficacy of drugs at safe concentrations. Moderate heterogeneity was observed (I²=64.2%, *P* < 0.001), with subgroup analyses identifying cell type, viral strain, genotype, drug concentration and intervention duration as significant influencing factors (*P* < 0.05). Intrinsic antiviral activity emerged as the primary source of heterogeneity. Quality assessment using BRISQ guidelines rated 15 studies as high quality and 17 as medium quality, while modified ROBINS-I classified 26 studies as low risk of bias and 6 as moderate risk. Publication bias was suggested by funnel plots and egger’s test. However, sensitivity analyses confirmed the robustness of the results.

**Conclusion:**

Forty-one of drugs and compounds demonstrate effective inhibition of PEDV replication in vitro, with antiviral efficacy influenced by experimental variables and predominantly determined by intrinsic antiviral activity. These findings offer valuable guidance for optimizing the design of PEDV antiviral studies and contribute insights for future in vivo efficacy and mechanistic research.

**Systematic review registration:**

Open science framework (10.17605/OSF.IO/V4C8Y).

## Introduction

Porcine Epidemic Diarrhea Virus (PEDV), a member of the genus *Alphacoronavirus* (*α*-CoV), is the primary etiological agent of Porcine Epidemic Diarrhea (PED) [1]. First identified in the UK in 1971,PEDV gradually across Europe, causing sporadic outbreaks throughout the latter half of the 20th century [2]. In 1973,China documented diarrhea cases resembling PEDV infection, with the virus formally isolated and identified in 1984 [3]. Since 2010, highly pathogenic PEDV variants have triggered large scale outbreaks in China and globally, posing a substantial threat to the global pig farming industry. Particularly in neonatal piglets, PEDV infection exhibits morbidity rates approaching 100% and mortality rates ranging from 80% to 100%, resulting in devastating economic losses to the breeding industry [4]. PEDV virions are spherical, measuring 95–190 nm in diameter and the virus possesses a single-stranded RNA genome approximately 28 kb in length [5]. The genome encodes replicase proteins (ORF1a and ORF1b), non-structural proteins, the auxiliary protein ORF3 and four structural proteins: spike(S), envelope(E), membrane(M), and nucleocapsid (N) [6]. Among these, the S protein is the primary envelope glycoprotein and plays a pivotal role in host cell infection [7]. It mediates viral entry and determines the virus’s host range, tissue tropism, cross-species transmissibility and trypsin dependent propagation [8].The *S* gene exhibits extensive genetic diversity and a high mutation rate, making it a critical marker for evaluating the virulence and genetic variability of field strains [9]. At present, despite widespread adoption of vaccination and biosecurity measures, the prevention and control of PED remain challenging. The high mutation rate and genetic diversity of PEDV significantly limit the protective efficacy of current vaccines, which fail to provide broad coverage against circulating strains [10]. Consequently, the development of effective antiviral drugs and intervention strategies to inhibit PEDV replication has become a research priority [11]. Recent advances in PEDV drug research have identified several promising candidates [12],including natural compounds such as quercetin [13, 14],curcumin [15] and berberine [16],as well as antiviral drugs like ribavirin [17]. Additionally, novel intervention strategies have shown inhibitory effects in vitro studies, including the GSK-3 inhibitor CHIR-99,021 [18], peptides M2 and M17 [19], the 3 C-Like protease inhibitor GC376 [20] and porcine IFN-λ3 [21]. However, significant heterogeneity in experimental conditions, drug dosages, and evaluation methods across studies hampers direct comparisons of efficacy and limits their clinical translation and application [22].

In response to the limitations of current research, this study aims to systematically evaluate the relative efficacy of various drugs and combination therapies in inhibiting PEDV replication in vitro through a systematic review and meta-analysis. By conducting a comprehensive literature search, rigorously screening study data, and performing quantitative analyses, this study seeks to elucidate the comparative effectiveness of different intervention strategies. The findings are expected to provide a robust theoretical foundation for the evidence-based prevention and control of PED and offer valuable guidance for future drug development and clinical applications.

## Methods

### Study search and inclusion criteria

To systematically review the application of chemical compounds in inhibiting PEDV infection, we conducted a comprehensive literature search using PubMed, Web of Science, and ScienceDirect databases. The search covered publications from the inception of each database to May 30,2025. The strategy employed a combination of the following keywords: “Porcine epidemic diarrhea virus”, “PEDV”, “Virus replication”, “Antiviral agents”, “Antiviral drugs” and “Natural compounds”. These keywords were chosen to focus on two primary themes: PEDV infection and antiviral compounds.

Study screening and evaluation were independently performed by two researchers to ensure objectivity and consistency. During the initial screening, titles and abstracts were reviewed to preliminarily determine whether the study addressed drugs and PEDV replication. In cases of disagreement, a third researcher was consulted to resolve conflicts and make a final decision on eligibility. Studies were included if they met the following criteria: (1) The full text was available in English and accessible for download. (2) Experimental data on the antiviral activity of compounds against PEDV were provided, with research limited to in vitro settings involving PEDV and mammalian cell lines or primary cells. (3) Interventions included either a single compound or a combination of drugs, with each experimental setup requiring a negative control group. (4) Outcome measures reported data on virus replication inhibition, expressed as TCID50, and included extractable means, standard deviations (SD), and sample sizes (n) from tables or figures.

### Exclusion criteria

To maintain analytical focus and ensure rigor in the study selection process, predefined exclusion criteria were applied. First, articles lacking complete data or full-text access were excluded. This included animal experiments, clinical trials, case reports, reviews, commentaries, conference abstracts, and studies missing essential information. Studies not related to PEDV or those focusing exclusively on inactivated viruses or pseudovirus particles were also excluded to preserve specificity within the scope of research.

Regarding intervention characteristics, studies that employed non-compound approaches or failed to explicitly identify the compounds investigated were excluded. Methodological issues, such as the absence of a negative control group, also led to exclusion. In addition, duplicate datasets published by the same research group across different journals were deemed ineligible to prevent over-representation of findings. Studies that did not report outcomes in terms of TCID50-based virus replication changes were excluded to ensure consistency and comparability of the outcome measures.

During data extraction, prioritization methods were implemented to focus on biologically significant outcomes. When multiple concentrations of a compound were tested, the concentration that exhibited the maximum observed effect was selected for inclusion. For studies comparing different PEDV strains, data were extracted separately for each strain to facilitate subgroup analyses based on viral strain type.

### Data extraction

To enhance clarity and facilitate analysis, data extracted from the included studies were systematically organized into a comprehensive table. This table incorporates essential elements for detailed comparison and pattern identification across studies. Key information includes the author’s details, publication date, cell type, and PEDV strain, providing contextual and methodological foundations. Furthermore, the table documents critical experimental parameters such as the drug name, intervention time, and concentration, offering insights into study design and treatment protocols. Viral replication outcomes are assessed through changes in TCID50, while key proteins involved in the drug’s mechanism of action are highlighted to elucidate the biological impact.

### Publication quality and bias analysis

To ensure the reliability and validity of the meta-analysis, a standardized approach was employed to comprehensively evaluate study quality and potential bias. First, methodological quality was assessed using a 7-item checklist adapted from the BRISQ (Biospecimen reporting for improved study quality) guidelines. The checklist focused on critical dimensions, including cell line authentication, mycoplasma contamination detection, biological replicates, experimental controls, standardization of key detection methods, data extractability, and cell source declaration. Studies were categorized into three quality levels based on their scores: low quality (0–2 points), moderate quality (3–4 points), and high quality (5–7 points).

Subsequently, potential bias was evaluated using a modified version of the ROBINS-I (Risk of bias in non-randomised studies of interventions) tool tailored to the context of in vitro studies. Modifications included the following:

1. Confounding factors control: Adapted to assess the consistency of experimental conditions, such as the standardization of cell lines, viral strains, and experimental methods.
2. Implementation of interventions: Modified to evaluate the procedural standardization of interventions, including drug concentrations, drug handling, administration methods, and control setup.
3. Outcome measurement: Adjusted to assess the standardization of TCID50 assays, focusing on culture conditions and interpretation criteria.
4. Data integrity: Evaluated to determine whether key data were comprehensively reported and extractable.

Studies were classified into three categories based on the risk of bias: low risk, moderate risk, and high risk.

1. Low risk of bias: Studies with clear reporting of cell line sources, confirmation of negative mycoplasma testing, consistent and controlled experimental conditions (e.g., cell lines, viral strains, drug concentrations), detailed explanations of intervention duration and negative control settings. TCID50 assay protocols must include clear culture conditions and interpretation criteria, and core data required for effect size calculations (e.g., means, standard deviations, and sample sizes) are fully extractable.
2. Moderate risk of bias: Studies where the core experimental design and data exhibit no major flaws, but secondary information is incomplete, posing a mild risk of bias. For example, studies may lack detailed reporting on cell line passage numbers or drug purity; however, critical experimental conditions remain standardized. TCID50 detection may lack detailed observation time points, but the culture conditions and interpretation criteria are clearly defined, and core data for effect size calculations are complete, with only minor auxiliary information missing that does not affect the reliability of results.
3. High risk of bias: Studies with significant methodological deficiencies, missing or poorly reported critical information. For example, studies may fail to report cell line sources, authentication results, or mycoplasma contamination testing. Experimental conditions may be inconsistent, with confounding factors uncontrolled. Drug intervention details may be unclear, or control group designs may be inappropriate. TCID50 assays may lack methodological rigor, and essential data for effect size calculations may be missing, rendering the key results unverifiable.

### Statistical analysis

In this study, rigorous statistical and methodological approaches were employed to evaluate differences in outcomes across eligible studies. The change in TCID50 was chosen as the primary outcome measure, with the SMD used to quantify effect sizes. Forest plots were generated to visually present the pooled effect estimates, accompanied by 95% confidence intervals (CIs) to indicate the precision of each measure. To assess and interpret study heterogeneity, the I² index was calculated, facilitating the identification of variability patterns and potential sources of inconsistency.

To evaluate the robustness of the findings, publication bias was analyzed using funnel plot visualization and egger’s regression test. Predefined subgroup analyses were conducted to explore the effects of key variables, such as cell type, compound type, viral strain, compound concentration, and intervention duration on the observed heterogeneity. These analyses provided deeper insights into how specific experimental conditions influenced the overall results.

Sensitivity analyses were subsequently performed using two complementary approaches. First, a leave-one-out approach systematically excluded individual studies to evaluate their influence on the pooled effect size. Second, a quality-stratified analysis was conducted, retaining only high-quality studies with BRISQ scores ≥ 5. Comparing the results of this stratified analysis with the full dataset allowed us to examine the impact of study quality on the final conclusions. All statistical analyses and data visualizations were performed using Stata software or the R programming language, ensuring accuracy and reproducibility.

## Results

### Study selection and characteristics

**Fig. 1:**
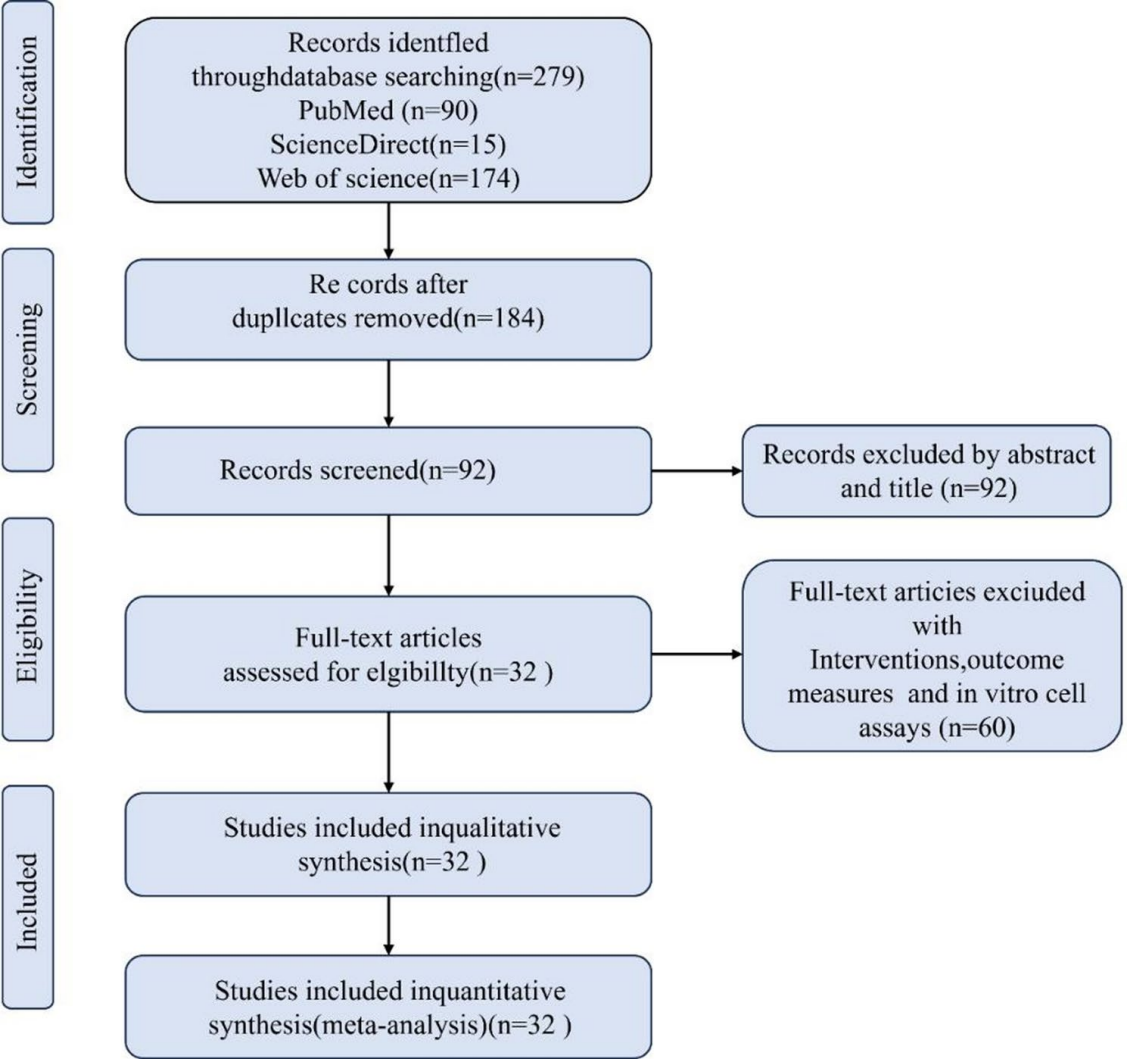
PRISMA flow diagram of study selection for the systematic review and meta-analysis. A total of 279 records were initially retrieved from PubMed (*n* = 90), Web of Science (*n* = 174), and ScienceDirect (*n* = 15). After removing 95 duplicate records, 184 studies were screened. At the title/abstract stage, 92 studies were excluded due to irrelevant topics or non-in vitro designs. A full-text evaluation of the remaining studies led to the exclusion of 60 articles due to mismatched interventions, outcome measures, or incomplete data. Ultimately, 32 eligible studies were included in both the qualitative synthesis and quantitative meta-analysis

To comprehensively evaluate the efficacy of drugs or compounds against PEDV infection, we conducted a systematic search across three databases (PubMed, Web of Science and ScienceDirect), initially identifying 279 potential studies. After removing 95 duplicate records, 184 studies were retained for title and abstract screening, of which 92 were excluded due to irrelevance to the core research theme. Subsequently, the remaining 92 studies underwent full-text assessment, and 60 were excluded for failing to meet the inclusion criteria (e.g., non-in vitro experiments, outcomes not reported as TCID50, or incomplete data). Ultimately, 32 studies satisfied all the screening criteria and were included in this systematic review and meta-analysis (Fig. 1). Table 1 summarizes the main characteristics of 32 studies, encompassing 987 independent biological assays conducted across three types of cell experiments. Among these, Vero cells were used in 31 studies, IPEC-J2 cells in six, and LLC-PK1 cells in one. Collectively, these studies investigated 41 drugs or compounds, all of which exhibited potential efficacy against PEDV infection. In addition, several key molecular targets were identified, including 3 C-like protease, PLP-2, RdRp, and p53, offering promising directions for the development of future antiviral therapies.

**Table 1:** Basic characteristics of the 32 studies included in the systematic review and meta-analysis

Studies	Cells	Samples	Strains	Genotypes	Interventions	Intervention concentrations(µM)	Intervention timess(h)	Key interacting protein	Quality score	Publication bias
Zheng Chen [23], 2021	Vero	21	CH/JXJA/2017	GII	Carbazole − 7/Carbazole − 18	40/60	24	unknown	6	Low
Mengfei Gong [24], 2023	Vero	96	DY/CV777	unknown/GI	Chrysin/Naringenin/Ribavirin	unknown	24/36/48	3 C-like protease/PLP-2	6	Low
Jixiang Liang [25], 2024	Vero/IPEC-J2	102	YN145	GII	Flavonol	80	24/26	Mpro	6	Low
Jieru Wang [26], 2024	Vero	15	AH2012/12	GII	Luteolin	25	unknown	pACE2	6	Low
Hongwei Xiang [27], 2024	Vero/IPEC-J2	24	AH-2018-HF1	GI	Berbamine	10	20	nsp3/nsp16	5	Low
Shijuan Dong [28], 2022	Vero	108	DR13	GI	Cepharanthine/Tetrandrine/Fangchinoline	20	36	unknown	6	Low
Zhonghua Li [29], 2025	Vero	24	YN13/DR13	GII/GI	Chebulinic acid	50	24	nsp5	5	Low
Qiancheng Jiang [30], 2025	Vero/IPEC-J2	12	LJX	unknown	Curcumin	64	unknown	JAK-STAT/ISG	6	Low
Kaiyuan Li [31], 2024	Vero	45	CV777	GI	Dehydroevodiamine/Ribavirin	6.25	12/48	MAPK/ERK1/2	4	Low
Limin Jiang [32], 2025	Vero	6	HM2017	GII	Ethyl caffeate	100	48	3 C-like protease	4	Some concerns
Yue Wang [33], 2023	Vero	54	DR13/HB	GI/GII	Ivermectin	3/6	28/25/24	p53	4	Low
Yue Zhang [34], 2021	Vero	24	HLJ	unknown	Hypericin	10	34	3 C-like protease	4	Low
Yue Wang [35], 2023	Vero/LLC-PK1	120	DR13/CV777/HNXX/HW/HB	GI/GII	Niclosamide	0.5/1/1.5	24	unknown	5	Low
Yaoying Jian [36], 2025	Vero	24	CH/JXJA/2017	GII	Indole alkaloid derivatives-14	2/10	24	unknown	5	Low
Jingping Ren [37], 2022	Vero	18	DR13	GI	Cinchonine	100	24	unknown	4	Low
Zhonghua Li [38], 2020	Vero	24	YN144/DR13	GII/GI	Quercetin	100	unknown	3 C-like protease	4	Some concerns
Pei Sun [39], 2022	Vero/IPEC-J2	24	AH-2018-HF1	unknown	Buddlejasaponin IVb	20	unknown	NF-kB	4	Some concerns
Wei Zeng [40], 2022	Vero	30	DR13/YN15	GI/GII	Levistolide A	60/80	36	unknown	4	Low
Pengcheng Wang [41], 2022	IPEC-J2	9	MS	unknown	ZINC12899676	10	24	NTPase	4	Low
Jun Wang [42], 2024	Vero	6	HM2017	unknown	Natural hyperoside	10	48	N/p53	4	Some concerns
Mingxia L [43], 2023	Vero	6	CH/HBXT/2018	unknown	Nicotinamide Efficiently	500	24	unknown	6	Low
Mingjun Su [44], 2023	Vero	6	CV777	GI	Octyl gallate	40	48	3 C-like protease	4	Low
Liang Li [45], 2024	Vero/IPEC-J2	24	AH-2018-HF1	GI	PA-824	30/50	21	p53	4	Low
Zi-Xin Huang [46], 2023	Vero	18	SHpd/2012	GI	Remdesivir	12	unknown	RdRp	5	Some concerns
Yiyi Hu [47], 2025	Vero	39	AH2012/12	unknown	Polyphenols-2/Polyphenols-5/Polyphenols-7/Polyphenols-17	0.5	24	unknown	6	Low
Jing Wan [48], 2022	Vero	24	CV777	GI	RAF265	10	24	S-Protein	4	Low
Pishun Li [49], 2024	Vero	18	CV777	GI	SP2509	1	36	LSD1	5	Some concerns
Hui-Jun Dong [50], 2018	Vero	12	CV777	GI	Homoharringtonine	0.5	48	unknown	4	Low
Pengcheng Wang [51], 2020	Vero	12	MS	unknown	Tomatidine	10	17	3 C-like protease	4	Low
Caiying Wang [52], 2025	Vero	12	LJX	unknown	Zn2+	160	24	PI3k-Akt-mTOR	4	Low
Hong-Jie Li [53], 2018	Vero	15	CH/ZMDZY/11	GI	LiCl	15,000	unknown	unknown	6	Low
Jieru Wang [54], 2023	Vero	15	AH2012/12	GII	Wogonin	100	48	3 C-like protease	4	Low
Total		987								

### Efficacy of drugs and compounds against PEDV infection in vitro

**Fig. 2:**
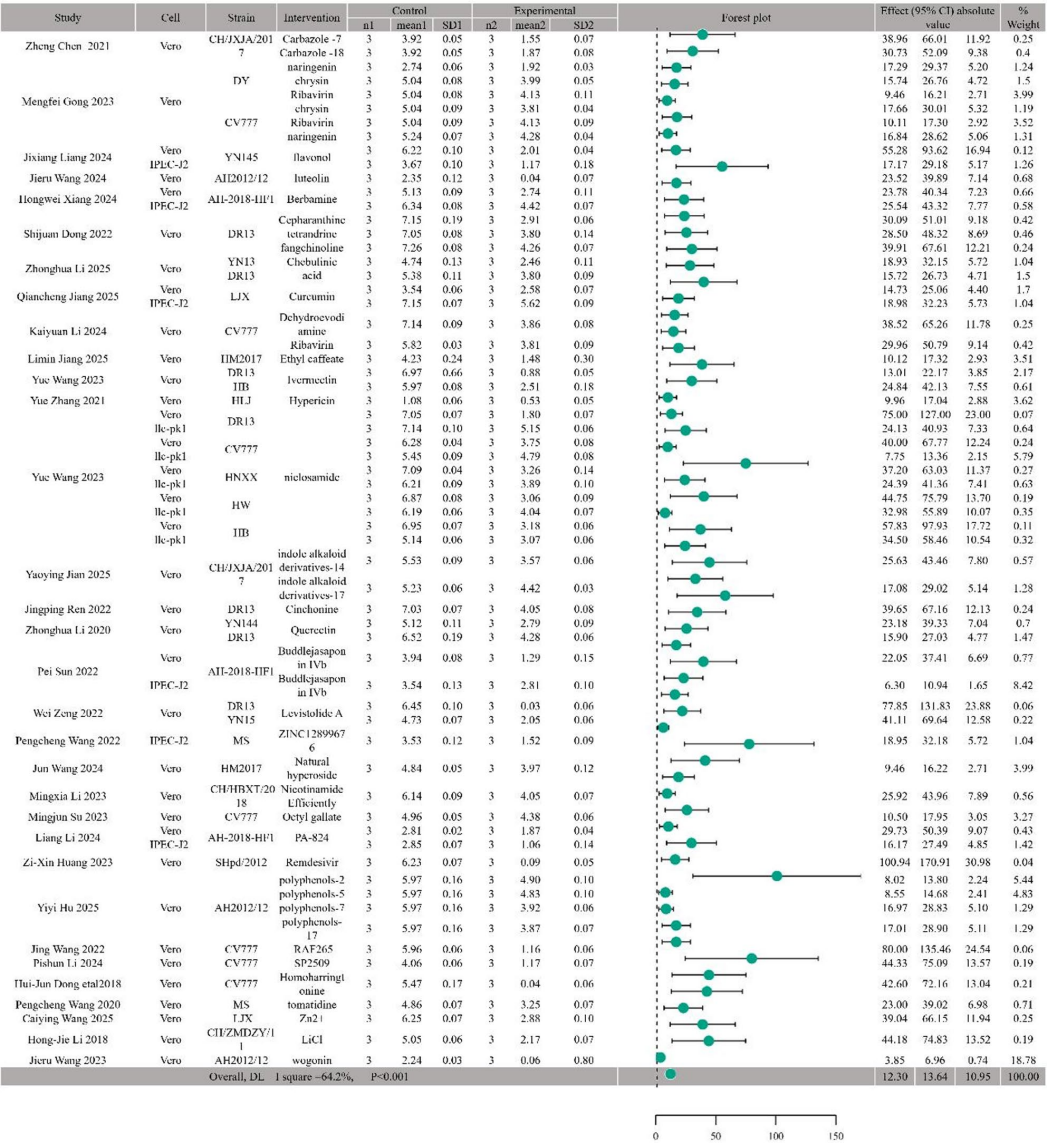
Forest plot of the in vitro antiviral efficacy of drugs against PEDV (random-effects model). Left table columns: study (first author and publication year), cell (cell model used), strain (strain used), intervention measures (tested drug/compound), control/Experimental (TCID50 values of untreated and drug-treated groups), SD1/SD2 (standard deviations of TCID50 in control and experimental groups), n1/n2(sample size). Forest plot elements: each square represents the SMD of individual studies, horizontal lines indicate 95% Cis, the diamond denotes the pooled SMD. Overall heterogeneity: I²=64.2%, *P* < 0.001, indicating moderate heterogeneity. Right table columns: effect (95% CI) (SMD and its 95% CI), weight (contribution of each study to the pooled result)

To evaluate the in vitro anti PEDV effects of 41 drugs under varying intervention concentrations and durations, we selected the change in TCID50 as the primary outcome measure and used the SMD to quantify effect sizes. Forest plots were generated using a random effects model in Stata MP17 software (I²> 50%, *P* < 0.001) to visually present the pooled effect estimates, accompanied by 95% CIs to indicate precision (Fig. 2).

Analysis of 32 pooled studies revealed that, compared to controls, the overall effect estimate for the anti-PEDV efficacy of the 41 drugs at safe concentrations yielded a SMD of -12.30 (95% CI: -13.64 to -10.95). Substantial heterogeneity was observed among the included studies (I²= 64.2%, *P* < 0.001).

### Study quality, publication bias and sensitivity analyses

To ensure the reliability and validity of the meta-analysis, we conducted a systematic quality assessment of the included studies across seven dimensions: cell authentication, detection of mycoplasma contamination, number of biological replicates, experimental control setup, standardization of critical detection methods, data extractability, and declaration of cell origin. Each criterion was scored on a scale of 1 point, with a maximum total score of 7 points. Studies were classified as low quality (0–2 points), medium quality (3–4 points), or high quality (5–7 points). Among the 32 studies evaluated, 17 were rated as medium quality and 15 as high quality (Table 1).

In addition, publication bias was assessed using the ROBINS-I tool based on five criteria: confounding control, intervention fidelity, outcome measurement, data integrity, and cell authentication. Results indicated that 26 studies had a low risk of bias, while 6 studies had a moderate risk. Publication bias was further analyzed using funnel plots and Egger’s test in Stata software. The funnel plot showed most data points concentrated near the central vertical line, suggesting high precision and low uncertainty in effect size estimates. However, a few data points scattered on the left with larger standard errors indicated some degree of publication bias. Egger’s test revealed a significant small-study effect (*P* < 0.05), which may indicate potential publication bias. For sensitivity analysis, two approaches were used. First, the exclusion of individual studies revealed that the majority of effect size estimates remained tightly clustered within a narrow range, demonstrating the robustness of the meta-analysis results. However, exclusion of the study by Jieru Wang [54] caused a deviation in the effect size, indicating this study’s relative sensitivity and lesser contribution to overall result stability. Second, we performed quality stratification analysis by retaining only high-quality studies (BRISQ score ≥ 5) for a new meta-analysis. This analysis yielded an overall effect size of -15.218 (95% CI: -17.12 to -13.31) while still indicating heterogeneity (I²= 54.3%, *P* < 0.05). Despite the observed heterogeneity, the exclusion of individual studies had minimal impact on the overall effect size, further demonstrating the stability and reliability of the meta-analysis outcomes.

### Sources of heterogeneity and subgroup analysis

Our meta-analysis of 32 studies revealed substantial heterogeneity (I²=64.2%, *P* < 0.001). To identify the factors contributing to this heterogeneity, we performed subgroup analyses focusing on key variables, including cell type, compound type, virus strain, compound concentration, and intervention duration. The results demonstrated that variations in cell type, strain type, genotype, drug concentration, and intervention duration significantly influenced effect sizes. Overall, moderate heterogeneity was observed (Table 2).

**Table 2:** Presents the criteria used for subgroup analysis, the number of studies included in each subgroup, the SMD estimates, the 95% CI, and the heterogeneity characteristics

Subgroup category	Number of included study	Number of included SMD	Standardized mean difference	95% Confidence interval		Heterogeneity index (%)	*P*-value
Cell							
IPEC-J2	6	6	-11.03	-14.66	-7.39	54.90%	0.05
LLC-PK1	1	5	-12.70	-17.55	-7.85	68.70%	0.012
Vero	31	52	-12.48	-14.00	-10.95	65.80%	< 0.001
Strain							
AH2012/12	3	6	-6.72	-9.09	-4.34	63.20%	0.018
AH-2018-HF1	3	6	-18.36	-27.31	-9.40	66.10%	0.012
CH/JXJA/2017	2	4	-23.40	-31.94	-14.86	NA	0.413
CV777	7	11	-12.90	-16.23	-9.57	64.90%	0.002
DR13	6	10	-24.52	-32.65	-16.40	45.90%	0.037
Genotype							
GI	17	29	-21.79	-26.40	-17.18	64.50%	< 0.001
GII	11	23	-20.36	-25.29	-15.43	71.30%	< 0.001
Drug concentration							
< 10µM	13	27	-19.96	-24.22	-15.69	60.70%	< 0.001
10µM-20µM	4	7	-25.93	-39.45	-12.41	76.20%	< 0.001
> 20µM	16	23	-21.11	-26.29	-15.93	71%	< 0.001
Time							
< 24 h	16	33	-22.52	-26.63	-18.40	56.60%	< 0.001
> 24 h	12	21	-15.63	-19.69	-11.57	66.30%	< 0.001
SMD							
Low	8	11	-7.25	-8.92	-5.58	NA	0.594
Moderate	17	30	-18.81	-21.27	-16.36	NA	0.999
High inhibitory effect	13	22	-40.61	-46.84	-34.38	NA	0.929
Total	32	63	-12.30	-13.64	-10.95	64.20%	< 0.001

To further elucidate the primary sources of heterogeneity, we conducted subgroup analyses based on the efficacy of different drugs in inhibiting PEDV infection, measured by SMD. Drugs were stratified into three categories based on their inhibitory effects. First, drugs with low inhibitory effects (SMD >-12) were represented by 8 studies and 11 SMDs, yielding a combined effect size of SMD = -7.25 (95% CI: -8.92 to -5.58). Second, drugs with moderate inhibitory effects (-30 < SMD ≤-12) included 17 studies, with a combined effect size of SMD = -18.81 (95% CI: -21.27 to -16.36). Finally, drugs with high inhibitory effects (SMD ≤-30) comprised 13 studies and 22 SMDs, resulting in a combined effect size of SMD = -40.61 (95% CI: -46.84 to -34.38). For all three groups, heterogeneity was low, indicating that the primary source of heterogeneity stemmed from differences in drug efficacy (Table 2).

Further analysis revealed that heterogeneity was influenced by the interplay between drugs, cell types, and virus strains, which were modulated by intervention duration and drug concentration. These interactions contributed to diverse inhibitory effects, significantly driving heterogeneity. Notably, combining drugs with low inhibitory effects (SMD > -12) and those with high inhibitory effects (SMD ≤-30) often increased overall heterogeneity. This pattern aligns with the sensitivity analysis, where sequential exclusion of studies highlighted the influence of individual studies on heterogeneity.

### Results of comparative antiviral efficacy of different drugs/compounds

To systematically analyze the efficacy differences of 41 drugs/compounds in inhibiting PEDV replication, this study sorted the compounds based on the SMD values and classified them into high efficacy, medium efficacy and low efficacy groups according to the efficacy intensity. The high efficacy group (SMD <-30) included 16 drugs, specifically Ribavirin, Carbazole-7, Carbazole-18, Flavonol, Cepharanthine, Fangchinoline, Dehydroevodiamine, Niclosamide, Cinchonine, Levistolide A, Remdesivir, RAF265, SP2509, Homoharringtonine, Zn²⁺ and LiCl. The pooled SMD of this group was − 40.61 (95% CI: -46.84, -34.38), indicating extremely strong inhibitory activity against PEDV replication. Among these, Remdesivir exhibited the most significant inhibitory efficacy, with a pooled SMD as low as -100.94 (95% CI:-170.91, -30.98).The medium-efficacy group (-30 < SMD<-12) contained 19 drugs, mainly natural products and partial synthetic compounds, namely Naringenin, Chrysin, Luteolin, Berbamine, Tetrandrine, Chebulinic acid, Curcumin, Ribavirin, Ivermectin, Indole alkaloid derivatives-14, Indole alkaloid derivatives-17, Quercetin, Buddlejasaponin IVb, ZINC12899676, Nicotinamide, PA-824, Polyphenols-7, Polyphenols-17 and Tomatidine. The pooled SMD of this group was − 18.81 (95% CI: -21.27, -16.36), showing moderate antiviral activity and favorable biosafety. Among these, Ribavirin had the optimal inhibitory efficacy in this group, with a pooled SMD of -29.96 (95% CI: -50.79, -9.14). The low efficacy group (SMD >-12) comprised 9 drugs, including Ribavirin, Ethyl caffeate, Hypericin, Buddlejasaponin IVb, Natural hyperoside, Octyl gallate, Polyphenols-2, Polyphenols-5 and Wogonin. The pooled SMD of this group was − 7.25 (95% CI: -8.92, -5.58), indicating a relatively limited inhibitory effect on PEDV replication. Among these, Octyl gallate showed the best inhibitory efficacy in this group, with a pooled SMD of -10.50 (95% CI: -17.95, -3.05). Notably, some drugs exhibited significant efficacy differences across different groups and their core influencing factors were closely related to experimental conditions and mechanisms of action. Ribavirin was present in all three high-, medium-, and low-efficacy groups. In the high-efficacy group, Ribavirin acted on the CV777 strain, with intervention at 100 µM for 12 h, exerting a potent antiviral effect by regulating the MAPK and ERK1/2 pathways. In the medium- and low-efficacy groups, Ribavirin acted on the DY strain, with the intervention time extended to 36 h and the concentration not specified; its targets shifted to 3 C-like protease and PLP-2. The altered targeting is presumably the key reason for the decreased efficacy. Buddlejasaponin IVb was present in both the medium- and low-efficacy groups. In both groups, the drug concentration was 20 µM, the intervention time was not specified, the acting strains were all genotype GI, and the antiviral effect was exerted by regulating the NF-κB pathway. However, differences in cell models were the core factor leading to efficacy differentiation. This drug showed better antiviral activity in Vero cells, which is presumably related to the lack of innate immune pathways in Vero cells and the more stable expression of drug targets. In contrast, in IPEC-J2 cells, the natural immune characteristics of intestinal epithelial cells may have weakened the drug’s effect.

## Discussion

In summary, this study systematically evaluated the inhibitory effects of 41 drugs and combined intervention strategies on PEDV replication, integrating data from 32 in vitro studies through a comprehensive systematic review and meta-analysis. The results revealed an overall effect size of SMD= -12.30, with a 95% CI of -13.64 to -10.95 at safe drug concentrations. This finding provided critical evidence based-insight for the efficacy evaluation of drug candidates in PEDV prevention and treatment.

This study observed significant heterogeneity (I²=64.2%, *P* < 0.001), highlighting the inherent complexity of in vitro antiviral research. Subgroup analysis indicated that factors such as cell type, viral genotype, drug concentration, and intervention duration significantly influenced the study outcomes, with the specificity of the drug effects being particularly prominent. Notably, we observed varying levels of heterogeneity across different cell models. Compared to LLC-PK1 and Vero cells, IPEC-J2 cells exhibited lower heterogeneity (I²=54.90%, *P* = 0.05), suggesting that IPEC-J2 cells are more suitable for investigating antiviral drug discovery against PEDV. This finding provided a foundation for further studies on the pathogenic mechanisms of PEDV in different cell models. IPEC-J2 cells, derived from porcine intestinal epithelium [55], differ significantly from Vero cells in terms of surface receptor expression and immune function [56]. Vero cells, originating from African green monkey kidney tissue, are non-immune cells and lack the unique immune regulatory mechanisms present in intestinal epithelial cells [57]. Therefore, this functional immune disparity may underlie the observed differences in drug efficacy across cell models [58]. However, it is important to note that the meta-analysis conducted in this study has yet to directly validate this hypothesis. We observed differences in antiviral drug sensitivity between highly pathogenic PEDV variants and classical strains. This phenomenon suggests that mutations in the viral S gene may be associated with variations in drug sensitivity. However, this association is based solely on speculative observations from subgroup analyses and has not been directly validated by the meta-analytic data presented in this study. Currently, it remains unclear whether mutations in the viral S gene directly mediate drug resistance, which specific mutation sites are involved in regulation, and the underlying molecular mechanisms. These questions require further targeted experimental investigation to be definitively addressed [59].Furthermore, among the 32 studies included in this analysis, 22 identified the molecular targets of the tested drugs, which encompass 3 C-like protease, the PI3K-Akt-mTOR signaling pathway, LSD1 protein, S protein, RdRp, p53 protein, N protein, the NF-κB signaling pathway, MAPK-ERK1/2 signaling pathway, JAK-STAT signaling pathway, and pACE2 protein, among others. The functional diversity of these targets within the viral life cycle and host cell regulatory processes underlies the significant variability in drug efficacy observed across studies, however, this result has not yet been supported by meta-analysis data and requires further investigation.

More importantly, the interaction between drug concentration and exposure time revealed that certain compounds exhibited cytotoxicity at high concentrations, whereas their antiviral efficacy weakened at lower concentrations. This heterogeneity reflects the dynamic interplay among PEDV, host cells, and drugs, emphasizing the need for standardizing experimental conditions to ensure reliable drug evaluations. When drugs were categorized based on their inhibitory effects, high-activity compounds (SMD <-30) were predominantly small-molecule inhibitors targeting viral RdRp. These drugs demonstrated well-defined mechanisms of action and consistently robust effect sizes. Medium-activity compounds (SMD between − 30 and − 12) were primarily natural products, while low-activity compounds (SMD >-12) were largely broad-spectrum antiviral agents developed in earlier studies, whose efficacy appeared highly sensitive to experimental conditions.

Quality assessment across seven methodological dimensions revealed that only 15 of the 32 studies met high-quality standards (scores ≥ 5). The most frequent shortcomings included unclear cell line origins and the absence of mycoplasma contamination screening. These findings highlight significant methodological deficiencies in current in vitro antiviral research, particularly regarding non-standardized cell models, which may lead to irreproducible results. Publication bias analysis using Egger’s test indicated the presence of a small-sample effect (*P* < 0.05). Sensitivity analysis further revealed that excluding the highly influential study by Wang et al. [54] altered the overall effect size, suggesting that single high-quality studies can disproportionately influence conclusions. These methodological limitations underscore the critical need for improved experimental rigor and standardization in future research.

Finally, it is important to acknowledge the limitations of this meta-analysis. First, the literature sources were restricted to three commonly used databases, which may have resulted in the omission of studies with significant findings. Not all studies were included in the subgroup analysis, and certain subgroups such as the LLC-PK1 cell subgroup and the CH/JXJA/2017 strain subgroup contained a limited number of studies, potentially introducing bias to the results.

This study has certain methodological limitations. Specifically, extracting only the maximum effect concentrations from multi-dose experiments precluded a comprehensive analysis of the complete dose-response relationships. This limitation may have resulted in an overestimation of the effect sizes for certain compounds. In this study, some studies contributed multiple effect sizes (e.g., from different strains, concentrations, or cell lines). However, we did not formally address the statistical dependence between these effect sizes in our analysis, which might violate the assumption of independence in meta-analysis. This limitation could potentially lead to an underestimation of the variability among effect sizes and an overestimation of the precision of our results.

It is necessary to clarify that this study uses the change in TCID50 as the primary outcome measure, which essentially quantifies the alteration in viral titer using the logarithmic dilution method. Highly effective antiviral drugs can lead to significant logarithmic reductions in viral titers, and such substantial differences in the raw data are reflected as large effect sizes in the calculation of the SMD. For the extreme SMD values observed in certain subgroups (e.g., SMD < -30), their biological significance can be interpreted as the corresponding drugs exhibiting potent inhibitory effects on PEDV titers. It should be emphasized that such large effect sizes are not statistical anomalies but rather a reasonable outcome resulting from the combination of TCID50, a sensitive indicator of viral titers, with the action of highly effective drugs. The core value of this result lies in its ability to provide a clear distinction in the antiviral efficacy of different drugs, thereby offering a quantitative basis for prioritizing drug candidates in further development.

Furthermore, all data were derived from in vitro models, and it is important to note that the replication mechanisms of PEDV in cell culture differ from those in porcine intestinal epithelial infections. In particular, the absence of an innate immune response in these models may lead to an overestimation of drug efficacy. Lastly, while the drugs tested were administered at safe concentrations, data on long-term toxicity are lacking, and the effects of high concentrations on the cell cycle remain insufficiently studied.

## Conclusions

In summary, numerous drugs and compounds have been shown to effectively inhibit PEDV replication in vitro, with their efficacy influenced by factors such as cell type, viral strain, genotype, drug concentration, and treatment duration. Notably, the primary determinant of antiviral activity is the intrinsic mechanism of action of the drugs. These findings provide valuable insights for optimizing experimental designs in PEDV antiviral research and lay the groundwork for further investigations into in vivo efficacy and underlying mechanisms.

## Data Availability

All data generated or analyzed during this study are included in this published article.The datasets used in the current study are available from the corresponding author upon reasonable request.
